# Editorial: The impact of microbially modified metabolites associated with obesity and bariatric surgery on anti-tumor immunity

**DOI:** 10.3389/fimmu.2023.1278862

**Published:** 2023-09-12

**Authors:** Yan Guo, Lin Qi, Ouyang Chen, Sharat Chandra, Dajin Zou

**Affiliations:** ^1^ Department of Endocrinology, Changhai Hospital, Naval Medical University, Shanghai, China; ^2^ Hunan Key Laboratory of Tumour Models and Individualized Medicine, The Second Xiangya Hospital, Central South University, Changsha, China; ^3^ Department of Cell Biology, Duke University Medical Center, Durham, NC, United States; ^4^ Department of Endocrinology and Metabolism, Punan Branch, Renji Hospital, Jiaotong University, Shanghai, China

**Keywords:** gut microbiota, metabolites, obesity, bariatric surgery, antitumor immunity

The human intestinal microbiota is regarded as the second set of the human genome and plays an important role in human metabolism, immunity, and the occurrence and development of tumors. The diversity of the human intestinal microbiota and its metabolites may affect the immune response in the tumor microenvironment (1). Obesity and its related chronic diseases, such as diabetes, cardiovascular disease and tumors, are closely related to the microbial environment of humans (2). With continuous weight loss and long-term metabolic benefits, bariatric surgery has become an important and common weight loss treatment in recent years (3). In this Research Topic, we collected articles examining the effects of bariatric surgery-induced changes in the bacterial microbiome and their metabolites on anti-tumor immunity. These articles can help clarify the relationship between intestinal microorganisms and their metabolites and the tumor microenvironment and treatment prognosis.

In the first article, Zhou et al. reviewed a series of studies on preclinical antitumor bacterial vaccines and new cancer combination therapies, focusing on the bacterial vaccines with the greatest chance of clinical application. Combining them with traditional therapies might successfully enhance the antitumor effect and allow us to understand clinical translation problems such as the treatment mode, biosafety, and production methods of bacterial vaccines. This is a strategy using external methods to translate microorganisms to exert antitumor benefits.

Our human body has a strong self-balancing and self-regulating ability and so it is possible that a change in intestinal microbiota caused by weight loss could also have anti-tumor benefits. Wang et al. have examined this possibility with evidence-based results. The characteristics of the intestinal microbiota and metabolic disorders caused by obesity may damage immune cells in the tumor microenvironment. Therefore, the favorable changes in intestinal microbial metabolites caused by bariatric surgery, such as short-chain fatty acids (SCFAs), inosine bile acid and spermidine, play an important role in anticancer immunity, providing a strategy to realize tumor immunotherapy by using supplementary microbial metabolites to simulate bariatric surgery.

Sun et al. focused on the changes in breast density caused by bariatric surgery, which is an independent risk factor for breast cancer and serves as an intermediate marker affecting the risk of breast cancer (4). They included a total of 535 people in 7 studies through meta-analysis. They found that, although bariatric surgery could reduce the risk of breast cancer, breast density increased significantly after bariatric surgery. The long-term effect of bariatric surgery on the risk of this type of obesity-related cancer, especially postmenopausal breast cancer, endometrial cancer, and colon cancer, remains a controversial topic (5). Whether it is related to the interaction between intestinal flora and host is worthy of further exploration.

Parida et al. found that enterotoxigenic *Bacteroides fragilis (B. fragilis)* colonized the intestines and breast ducts and secreted *B. fragilis* toxin, which accelerated the early spread of breast cancer cells from the primary tumor, caused systemic inflammation, and promoted the development of metastatic lesions by changing the tumor immune microenvironment. *B. fragilis* is a common intestinal colonization bacterium that is associated with obesity in children and adolescents (6). Colonization of *B. fragilis* in mice fed a high-fat diet can lead to more severe metabolic dysfunction and hyperglycaemia (7). Therefore, if the work in this contribution proves correct, a reduction in the colonization of the intestine with *B. fragilis* following bariatric surgery might be exploitable to reduce early metastasis of breast cancer to the liver and lungs.

Chen et al. further explored the correlation between the metabolic activity of breast cancer cells and changes in the intratumor microbiota, analyzed the standardized intratumor microbial abundance data of 1085 breast cancer patients and 32 single-cell RNA sequencing samples, and comprehensively described the relationship between intratumor microorganisms and metabolism, suggesting the potential role of some metabolism-related microorganisms in prognosis and the tumor immune microenvironment. For example, patients with high Campylobacter abundance and inositol phosphate metabolic activity have the worst prognosis, indicating the potential value of correcting microbial disorders in cancer treatment.

Chen et al. established a mouse model of type 2 diabetes mellitus complicated with hepatocellular carcinoma (T2DM+HCC) and fed *Lactobacillus brevis*, which is recognized as a probiotic that can improve blood glucose and body weight (8). Their studies have confirmed that, regardless of the concentration, *Lactobacillus brevis* can effectively improve blood glucose levels and insulin resistance in T2DM+HCC mice as well as pathological and inflammatory/metabolic indices of the liver and pancreas, such as total bile acid (TBA), lipopolysaccharides (LPS) and trimethylamine oxide (TMAO). This shows the potential antitumor effect of probiotic intervention on T2DM+HCC.

In conclusion, the articles included in this Research Topic significantly enhance our understanding of the important role of intestinal microorganisms and their metabolites associated with weight loss and metabolic improvement in tumorigenesis and metastasis to improve the effect and prognosis of cancer immunotherapy. They contribute to a better understanding of: (i) bacterial-mediated tumor immunotherapy, which may be an effective treatment for cancer patients, (ii) the effects of microbial metabolites associated with obesity and bariatric surgery on the characteristics and prognosis of certain tumor types (such as breast cancer), (iii) the relationship between microbiota characteristics and host metabolic heterogeneity in solid tumors such as breast cancer, and (iv) the palliative effect of probiotics in the codisease of metabolic diseases and malignant tumors that reveals the possibility of exerting antitumor effects by regulating metabolism.

## Author contributions

YG: Writing – original draft. LQ: Writing – review & editing. OC: Writing – review & editing. SC: Writing – review & editing. DZ: Writing – review & editing.
